# Supplementary material for: A Humanized Yeast Phenomic Model of Deoxycytidine Kinase to Predict Genetic Buffering of Nucleoside Analog Cytotoxicity
Source: Genes (Basel). 2019 Sep 30;10(10):770. doi: 10.3390/genes10100770 (PMC6826991; doi:10.3390/genes10100770)
Supplement: Supplementary file 1 [file genes-10-00770-s001.zip › Additional_Files/Additional_File7_GOTermHeatmaps.zip!/Additional_File7_GOTermHeatmaps/RNA_localization.pdf]

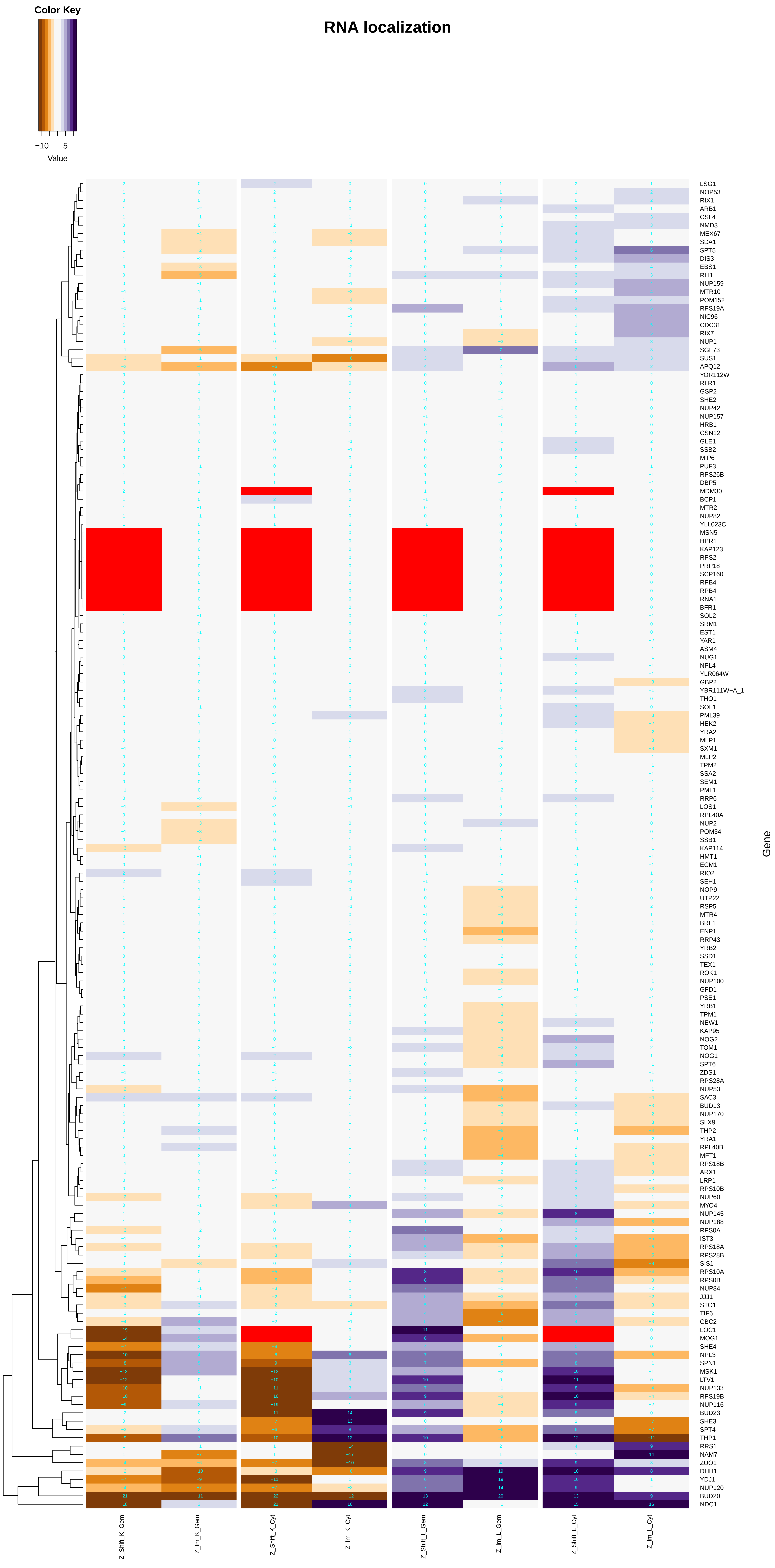
RNA localization
Color Key
−10 5
Value
Gene
LSG1
NOP53
RIX1
ARB1
CSL4
NMD3
MEX67
SDA1
SPT5
DIS3
EBS1
RLI1
NUP159
MTR10
POM152
RPS19A
NIC96
CDC31
RIX7
NUP1
SGF73
SUS1
APQ12
YOR112W
RLR1
GSP2
SHE2
NUP42
NUP157
HRB1
CSN12
GLE1
SSB2
MIP6
PUF3
RPS26B
DBP5
MDM30
BCP1
MTR2
NUP82
YLL023C
MSN5
HPR1
KAP123
RPS2
PRP18
SCP160
RPB4
RPB4
RNA1
BFR1
SOL2
SRM1
EST1
YAR1
ASM4
NUG1
NPL4
YLR064W
GBP2
YBR111W−A_1
THO1
SOL1
PML39
HEK2
YRA2
MLP1
SXM1
MLP2
TPM2
SSA2
SEM1
PML1
RRP6
LOS1
RPL40A
NUP2
POM34
SSB1
KAP114
HMT1
ECM1
RIO2
SEH1
NOP9
UTP22
RSP5
MTR4
BRL1
ENP1
RRP43
YRB2
SSD1
TEX1
ROK1
NUP100
GFD1
PSE1
YRB1
TPM1
NEW1
KAP95
NOG2
TOM1
NOG1
SPT6
ZDS1
RPS28A
NUP53
SAC3
BUD13
NUP170
SLX9
THP2
YRA1
RPL40B
MFT1
RPS18B
ARX1
LRP1
RPS10B
NUP60
MYO4
NUP145
NUP188
RPS0A
IST3
RPS18A
RPS28B
SIS1
RPS10A
RPS0B
NUP84
JJJ1
STO1
TIF6
CBC2
LOC1
MOG1
SHE4
NPL3
SPN1
MSK1
LTV1
NUP133
RPS19B
NUP116
BUD23
SHE3
SPT4
THP1
RRS1
NAM7
ZUO1
DHH1
YDJ1
NUP120
BUD20
NDC1
Z_Shift_K_Gem
Z_Im_K_Gem
Z_Shift_K_Cyt
Z_Im_K_Cyt
Z_Shift_L_Gem
Z_Im_L_Gem
Z_Shift_L_Cyt
Z_Im_L_Cyt

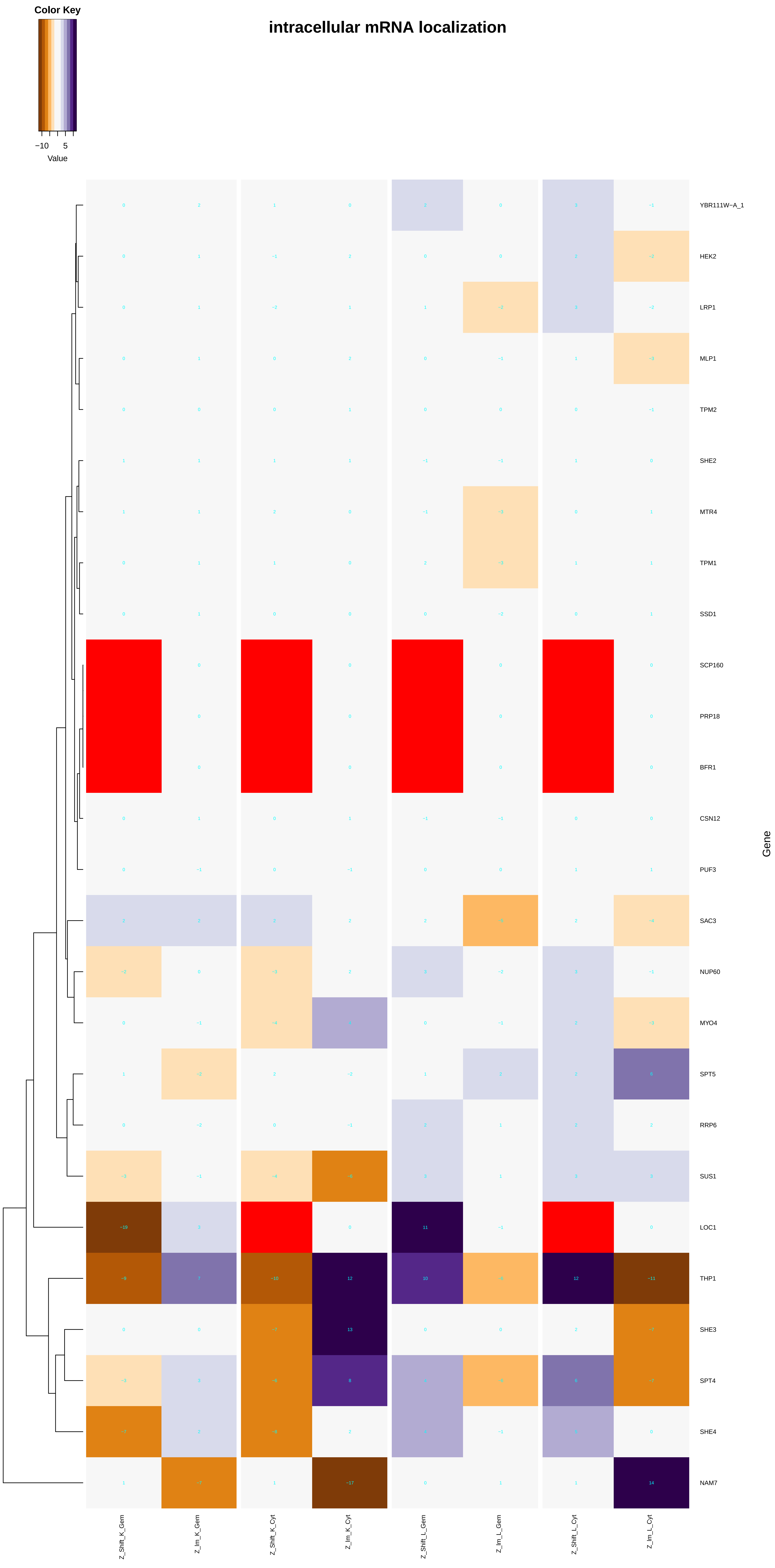
Color Key
−10
5
Value
intracellular mRNA localization
YBR111W−A_1
HEK2
LRP1
MLP1
TPM2
SHE2
MTR4
TPM1
SSD1
SCP160
PRP18
BFR1
CSN12
PUF3
SAC3
NUP60
MYO4
SPT5
RRP6
SUS1
LOC1
THP1
SHE3
SPT4
SHE4
NAM7
Gene
Z_Shift_K_Gem
Z_Im_K_Gem
Z_Shift_K_Cyt
Z_Im_K_Cyt
Z_Shift_L_Gem
Z_Im_L_Gem
Z_Shift_L_Cyt
Z_Im_L_Cyt

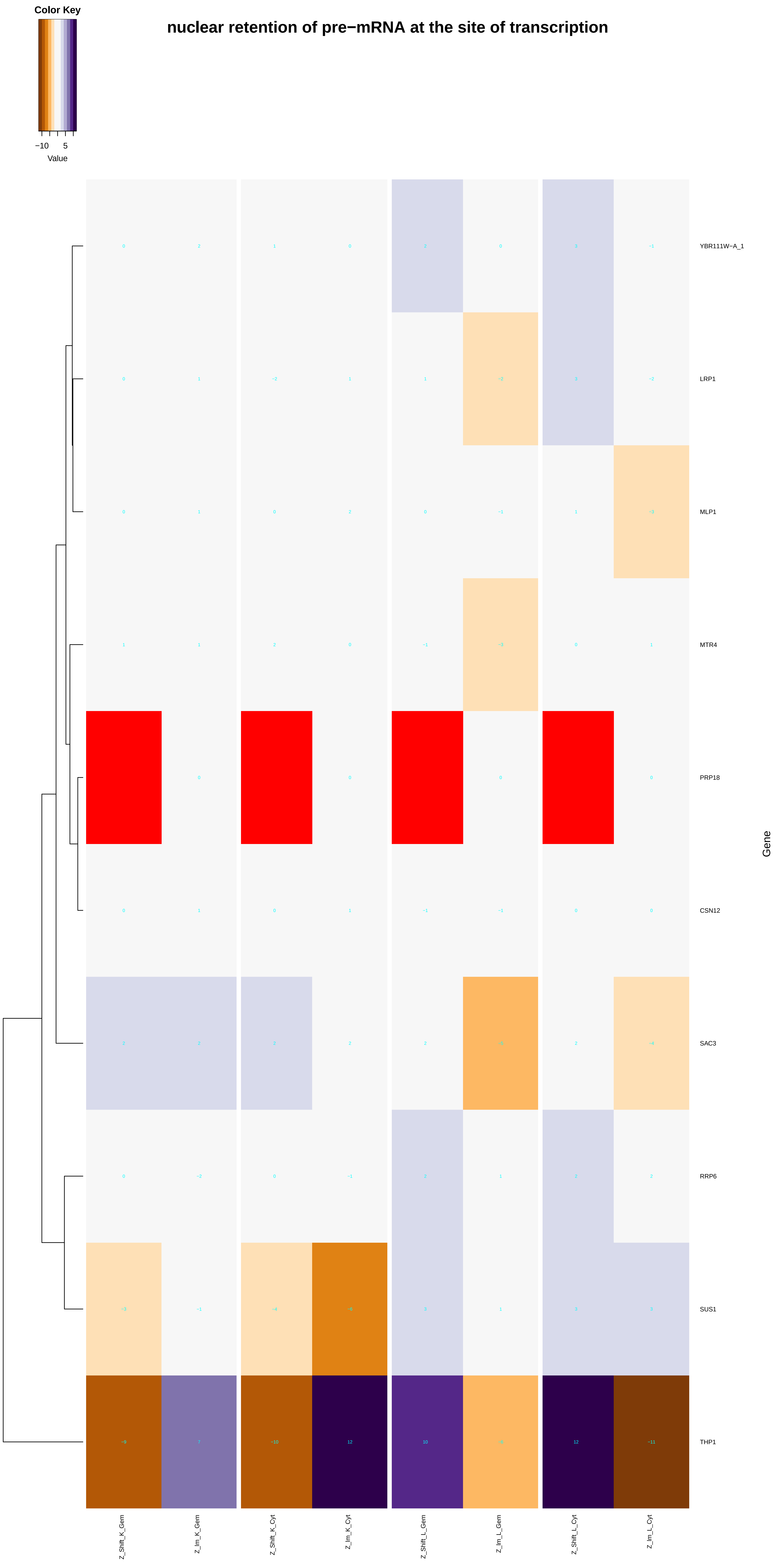

Color Key
−10
5
Value
nuclear retention of pre−mRNA at the site of transcription
YBR111W−A_1
LRP1
MLP1
MTR4
PRP18
CSN12
SAC3
RRP6
SUS1
THP1
Gene
Z_Shift_K_Gem
Z_Im_K_Gem
Z_Shift_K_Cyt
Z_Im_K_Cyt
Z_Shift_L_Gem
Z_Im_L_Gem
Z_Shift_L_Cyt
Z_Im_L_Cyt
